# Prebiotics: Ignored player in the fight against cancer

**DOI:** 10.1002/cnr2.1870

**Published:** 2023-07-17

**Authors:** Parichita Mishra, Vidhi Manish Badiyani, Sakshi Jain, Sruti Subramanian, Sakshee Vinay Maharaj, Ashwini Kumar, Bhisham Narayan Singh

**Affiliations:** ^1^ Department of Ageing Research, Manipal School of Life Sciences Manipal Academy of Higher Education Manipal Karnataka India; ^2^ Biotechnology and Bioinformatics Area NIIT University Neemrana Rajasthan India

**Keywords:** cancer, microbiome, prebiotics, tumour

## Abstract

**Background:**

Prebiotics is a relatively neglected area in cancer research, despite evidence suggesting that it plays a key role in suppressing tumour growth and improving immune function.

**Recent Findings:**

Including prebiotics in the diet has been shown to strengthen the immune system and can better slow down or prevent the growth of tumours. It has also been strongly indicated in various scientific studies that prebiotics can contribute to the sustenance of a healthy microbiome, which in turn plays an important role in increasing the effectiveness and reducing the side effects of cancer treatments.

**Conclusion:**

In the present review article we highlight the mechanisms by which prebiotics like inulin, fructooligosaccharide (FOS), β‐glucan, pectin, and xylooligosaccharide (XOS) function. Furthermore, the beneficial effect of incorporating prebiotics during cancer therapy to improvise gut health and prevent/reverse the damage caused to patients due to chemotherapy has also been elaborated.

## 1 INTRODUCTION

**FIGURE 1:**
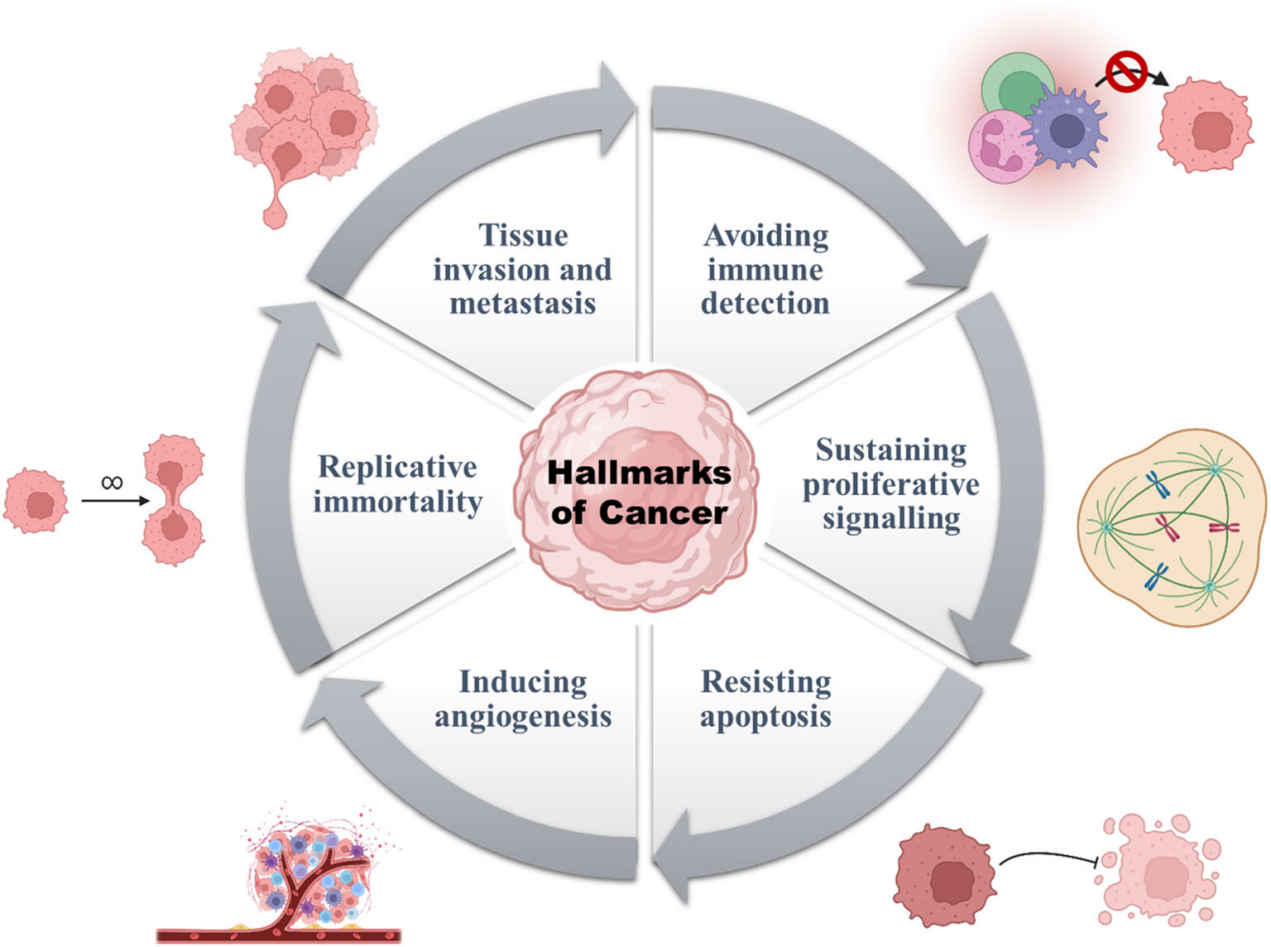
The important hallmarks of cancer.

Cancer is a global health problem that has affected over 18.1 million people worldwide, as per a study conducted in 2020.^1^ Age also plays a key role in cancer occurrence as the incidence increases significantly after 60 years of age, constituting more than 75% of the total affected population.^2^ However, it must be noted that children and adolescents are susceptible to some types of cancers, like retinoblastomas and bone cancers.^2^ Cancers can be broadly understood by checking a list of hallmarks that are acquired due to various factors such as—lifestyle, environment, genetic predisposition, and so on.^3^ These hallmarks were accurately summarised by the scientists Douglas Hanahan and Robert Weinberg and has been illustrated in Figure 1.

One of the many approaches taken by clinicians and scientists in designing treatment plans for patients with cancer has been to keenly study the tumour microenvironment. This has helped in understanding the pathophysiology of cancers, as not all progress at the same rate.^3^ Also, cancers are often caused by the accumulation of multiple mutations to oncogenes and tumour‐suppressor genes.^3^ Treatment modalities for cancers span across a wide array of interdisciplinary fields. Some popular treatment options are chemotherapy, radiotherapy, surgery, and targeted therapy using monoclonal antibodies and small molecules.^4^ However, these treatment modalities are often plagued by dangerous side effects like peripheral neuropathy, anaemia, formation of edemas, hair loss, chronic fatigue, thrombocytopenia, and delirium, just to name a few.^5^ They can drastically affect the quality of life in patients, and thus, treatment using prebiotics has seen a surge in popularity. Prebiotics are fermented compounds and were introduced in 1995 by the scientists Glenn Gibson and Marcel Roberfroid.^6^ Prebiotic compounds help in the growth of healthy bacteria in the gut and permit certain changes in the creation, structure, and function of gastrointestinal microflora, thus aiding in numerous profits to the hosts.^7^ The human gut is home to millions of microorganisms, collectively known as the gut microbiome. The gut microbiome is essential to human health by aiding digestion, producing essential vitamins and minerals, and regulating the immune system.^8^

Prebiotics contain certain non‐digestible substances like pectin, starch, galactooligosaccharides, and inulin which promote the growth of favourable bacteria present in the gut for better results in anti‐cancer treatment. Gibson and Roberfroid were the first to implement the concept of prebiotics as a stimulant to bring fermentation into the human microbiome.^9^ Prebiotics are believed to aid the formation of small‐chain fatty acids (SCFAs) and contribute to the reduction of products that come from protein degradation. They also exert signals that subdue gastrointestinal carcinogenic effects and have an anti‐adhesive nature against pathogens.^10^, ^11^

## 2 PREBIOTICS AND THEIR HEALTH BENEFITS

**FIGURE 2:**
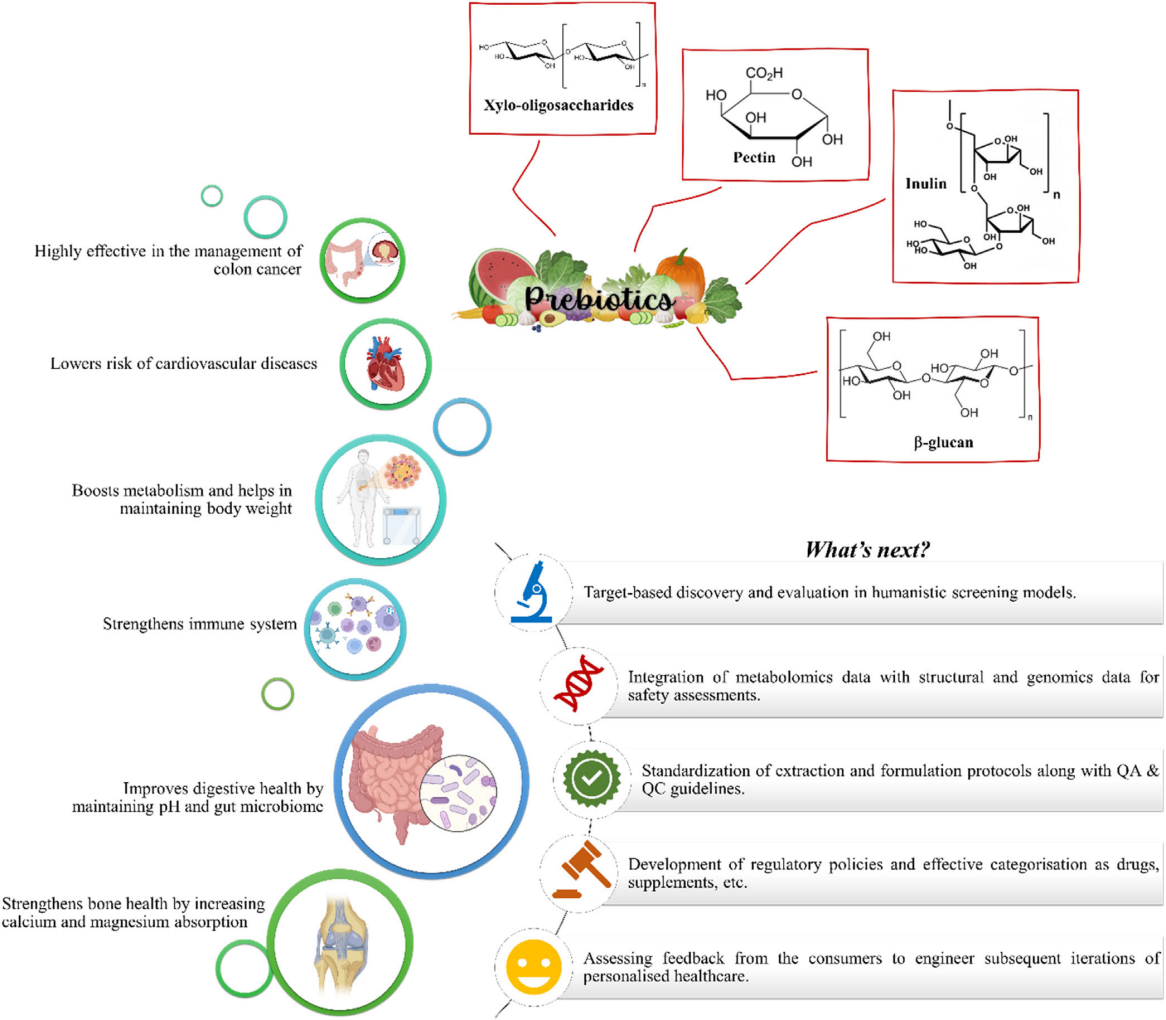
An overview of the health benefits of prebiotics and a discussion of future perspectives.

Recent research has shown that prebiotics and non‐digestible dietary fibres stimulate the growth and activity of beneficial microorganisms, especially gut bacteria. These compounds resist digestion in the upper gastrointestinal tract and are fermented by colonic bacteria in the lower gastrointestinal tract, leading to the production of short chain fatty acids which have various physiological effects such as—they can act as energy sources for intestinal microbiota and other host cells, contribute to signalling mechanism, which further shapes the environment for gut microbiome. Prebiotics also play an important role in modulating gut microbiota composition and metabolic activity by promoting gut health and reducing inflammatory responses. Common examples of prebiotic compounds include inulin, fructooligosaccharides, beta‐glucan, pectin, xylooligosaccharides, and so on, which can be found in various plant‐based foods such as vegetables, fruits, grains, legumes,^12^ as shown in Figure 2.

Inulin and mucin were tested for the potential to inhibit melanoma growth in syngenic mice models.^13^ Both of these prebiotics influenced the changes in the mice model's gut microbiota by increasing the population of *Bacteroides* spp. and *Akkermansia muciniphila*.^13^ Introduction of inulin and mucin in the feed also enhanced the expression of key anti‐tumour immunoregulatory genes linked to antigen presentation and chemokine production in tumour.^13^

The fermentation process yields a lot of essential products like short chain fatty acids (SCFA), essential vitamins, enzymes that help in disease resistance and immune response mechanism. They are produced by the gut microbiota during the fermentation of partially nondigestible polysaccharides that are absorbed as a source of daily energy requirements in humans. The highest amount of SCFAs is found in the colon. SCFAs constitute specific bacteria like *Bifidobacterium* sp. and *Lactobacillus* sp. The growth and proliferation of intestinal epithelium are generated by elements of SCFA—acetate, propionate, and butyrate. Butyrate is a distinct SCFA produced by gut microbes and acts as a good anti‐inflammatory agent by improving immunomodulation.^14^, ^15^ Apart from this, butyrate has many other positive effects on the body, including decreasing the risk of aetiology of colon cancer and adenoma development, reducing oxidative stress, detoxifying electrophilic products, and inducing enzymes for protection against carcinogens.

The human microbial system comprises trillions of microbial cells that share a symbiotic relationship in the company of numerous hosts in the human body, like gut bacteria. The presence is a primary indicator of one's well‐being. Recent studies have shown that variation in the gut microbiome can result in dysregulation of the immune system and is found to be a cause of autoimmune diseases. There are also microbiomes that are found to be beneficial to health. After extensive research probiotic bacteria like *Lactobacillus* sp. and *Bifidobacterium* sp. have been found to be valuable to gut health as well. These gut microbiomes play a massive role in building good immunity as they integrate with the host cells and regulate immune homeostasis. The presence of microbial cells is associated with the growth of different cancers in the epithelial barrier and sterile tissues.^6^, ^7^ Some common health benefits associated with long‐term prebiotic consumption have been discussed below.

### 2.1 Enhanced immune function

The gut microbiota also plays a crucial role in developing and regulating the immune system. Beneficial gut bacteria can help regulate the immune response and protect against infections. Prebiotics can help promote the growth of beneficial bacteria in the gut, improving immune function and reducing the risk of infections. It has been observed that prebiotics can increase the production of immunoglobulins, which are antibodies that help to fight off infections.^16^, ^17^

### 2.2 Reduced inflammation

Inflammation is a key factor in developing and progressing many chronic diseases, including autoimmune disorders, cancer, and heart diseases. Prebiotics have been shown to have anti‐inflammatory effects by promoting the growth of beneficial bacteria in the gut and reducing the production of pro‐inflammatory cytokines. As a result, prebiotics can reduce inflammation in the gut and throughout the body, which may help to reduce the risk of cancer development and progression.^16^, ^18^

### 2.3 Improved heart health

Prebiotics can help reduce cholesterol levels and lower blood pressure, which are important factors in maintaining good heart health. This is because prebiotics stimulates the growth of beneficial bacteria in the gut microbiome, which in turn produce compounds that help regulate cholesterol levels and blood pressure.^19^

### 2.4 Better weight management

Obesity is a major public health concern and is associated with an increased risk of many chronic diseases, including type 2 diabetes, cardiovascular disease, and certain types of cancer. The gut microbiota has been implicated in the development and regulation of body weight and metabolism, and prebiotics have been shown to have potential anti‐obesity effects. Recent studies have found that prebiotics can help to reduce the risk of obesity by promoting the growth of beneficial bacteria that are associated with a healthy weight. Prebiotics can increase the production of SCFAs, which can reduce inflammation and improve insulin sensitivity, potentially preventing the development of metabolic disorders and reducing the risk of obesity.^20^, ^21^

### 2.5 Improved digestion

Prebiotics can help regulate bowel movements and reduce constipation. This is because prebiotics are not digested in the small intestine but instead reach the colon intact, which has a high population of beneficial bacteria. These beneficial bacteria produce short‐chain fatty acids, which help regulate bowel movements and promote digestion.^16^

Although, prebiotics individually have certain antitumor effects, along with exercise they have unitedly exhibited positive immune responses against the tumour microenvironment, as shown in Table 1. An FMT study on breast cancer was executed for the same which focused on how the gut microbiomes responded to oligofructose (a prebiotic fibre) in addition to exercise. Two groups of pre‐exercise participants and post‐exercise participants were compared. It was observed that the post‐exercise group which was treated with oligofructose showed the lowest tumour volume and the highest amount of rehabilitation.^28^ This study can be helpful to obese patients.^28^ Spencer et al discovered the importance of combining prebiotics with diet. He concluded that prebiotics would contribute to a positive change in patients with melanoma.

**TABLE 1:** A summary of the effects of various prebiotic compounds on colon cancer, melanomas, breast cancer, lung cancer, and prostate cancer.

Type of cancer	Compound	Target	Effects and mechanism of action	References
Colon cancer	Raftilose/FOS (fructooligoscharide)	Cyclooxygenase‐2 (COX‐2), GST‐P and i‐NOS	Reduced the expression of i‐NOS and GST‐P in colon cancer‐inducing tumours and decreased azoxymethane (AOM) induced colon cancer.	22
Colon cancer	Pectin	Galectin‐3 and TLR4	Increased caspase‐3 activity and apoptosis observed. DNA fragmentation is directly proportional to apoptotic cells.	23
Metastatic melanoma	Inulin	K‐ras and N‐ras in CD4+ and CD8+ T‐helper cells	Increased the relative abundance of *Bacteroides* spp. led to the inhibition of BRAF mutant melanoma growth and enhanced the efficacy of a MEK inhibitor against melanoma.	13
Breast cancer	Polyphenol	Breast cancer (BRCA) gene	Increased levels of bacteria (*Bifidobacterium* sp. and *Lactobacillus* sp.) and SCFAs which resulted in decreased primary tumour growth and metastases and decreased 4T1 breast tumour metastasis through inhibited macrophage infiltration and angiogenesis.	24
Colorectal cancer	Human milk oligosaccharides.	IL‐6, IL‐1β, and TNF‐α	Regulation of epidermal growth factor receptor (EGFR) signal pathways resulted in growth arrest of intestinal cells. This significantly inhibited cell proliferation.	25
Colorectal cancer	β (1–4) galactooligosaccharides	Aberrant crypt foci (ACF)	Increased abundance of *bifidobacteria*, resulting in inhibition of ACF formation n reduction of DMH‐induced ACF.	25
Prostate cancer	Pectin	LNCaP and LNCaP C4‐2	Increased apoptosis and activation of caspase‐3 in both cell lines.	23, 26
Prostate cancer	Modified citrus pectin (MCP)	Galectin‐3	Decreased Gal‐3 expression by 80% in the nucleus and simultaneously increased its expression in the cytoplasm, significantly reduced the invasive and migratory potential of PCa cells.	27

## 3 PREBIOTICS IN CANCER GROWTH, IMMUNITY, AND THERAPY

Recently, the effects of prebiotics on cancer have been a major topic of research and discussion. Studies could suggest that intake of specific prebiotics like fructo‐oligosaccharides also known as fructans might be beneficial in decreasing the risk of colon cancer. Prebiotics such as inulin, oligofructose, fructooligosaccharides (FOS), galactooligosaccharides (GOS), soybean oligosaccharides, and resistant starches can also have a beneficial impact on mineral absorption and metabolism. The relationship between prebiotic‐mineral absorption and anti‐cancerous activity is complex and involves several factors. Prebiotics can increase the solubility of minerals in the gut by producing chain fatty acids (SCFAs). This, in turn, can lead to the formation of SCFA‐salt compounds that make minerals more easily absorbed. Prebiotics also help to increase the surface area available for mineral absorption, promote the expression of calcium‐binding proteins, and break down the phytic acid‐mineral complex, which releases bound minerals. Additionally, prebiotics can release bone‐regulating substances like phytoestrogens found in certain foods, stabilise gut bacteria and mucus, and help maintain a healthy gut microbiome.^29^

A study by Femia in 2002 was based on prebiotic Raftilose Synergy 1 which is primarily inulin that has been supplemented with oligofructose. F344 rats were used as a model to execute this study. The variation in expression of glutathione S‐transferase Pi (GST‐P) and cyclooxygenase 2 (COX2) genes was examined in normal, and tumour‐induced animals by stimulating them with prebiotics. Results showed that the rats with a dosage of prebiotics showed a reduced expression of GST‐P compared to those who were not treated with any kind of prebiotics. A homogenous effect was observed in the case of tumours. They also concluded that prebiotics successfully reduced the expression of inducible nitric oxide synthase (i‐NOS) in the tumours of rats. The above results suggest that prebiotics reduce induced carcinogenesis and have a protective characteristic towards the host's body.^22^ The mechanism of alteration of gut microbiota by prebiotics happens over a period of a few weeks however, the precise mechanism by which prebiotics work is yet to be fully understood. Although the variations may subside after a short period of resuming normal diet intake, the treatment still works in medical conditions. Recently a clinical trial was conducted on patients with gynaecological cancer. It was evident that prebiotics takes approximately 1–2 weeks to function, and the effects are short‐lived, but impactful.^30^

Cancer immunotherapy is one of the most advanced developments in the field of cancer cure led by immune checkpoint blockers. Immune checkpoint blockers obstruct the production of checkpoint proteins secreted by T‐cells, B‐cells, and some cancer cells. These inhibitors can rehabilitate T‐cells, which have been rendered inactive by tumour cells, allowing them to respond to antigens again.^29^ Inulin, an eminent prebiotic supplement, appeared to diminish the growth of transplantable melanoma in T‐cell dependent model. A sharp decline in tumour development was observed in rat models when they were fed a diet supplemented with inulin and oligofructose which also improved their survival.^33^, ^34^

Prebiotics can improve the host's immunity by altering the gut microbiota responsible for producing immune responses against emerging tumours. Diet is one of the strongest modulators of bacteria residing in the gut. This is a key aspect in all types of cancers because both innate and adaptive immunity is believed to be key in blocking cancer development by preventing tumour cell metabolism and enhancing the activity of anti‐cancer T‐cells. CD8+ T‐cells are one of the most important elements in cancer control and play a major role in subsiding cancer growth. T‐cells are the main target in cancer treatment. Thus, to prevent cancers, variation in the properties of gut microbiota with the help of nutritional mediation, specifically by means of prebiotics serves as a treatment promoter. It is observed that doses of inulin incorporated in the diet has modulated gut microbiomes positively by revealing strong anti‐tumour activity. A study based on inulin‐mediated diet indicated activation of targeted T‐cells in epithelia tissues and anti‐tumour immune responses.^33^ Microbial metabolites induce certain signals that strike immune responses through a systematic circulation. This controls tumour progression and immune responses. Gut microbiota persistently mediate immune response according to their composition, which results in modifying innate as well as adaptive immunity at various levels. Innate immunity has a protective response towards all antigens.

Prebiotics can also activate specific signalling pathways in immune cells, leading to either pro‐ or anti‐inflammatory responses. Prebiotics like inulin, short chain galactooligosaccharides/long chain fructooligosaccharides (scGOS/lcFOS) and *Platycodon grandiflorum* have been shown to induce an immunosuppressive environment by promoting fork head box protein 3 gene (FOXP3) gene expression and Interleukin 10 (IL‐10) secretion, while other prebiotics like a3‐sialyllactose and FOS can inhibit proinflammatory cytokine expression, creating an anti‐inflammatory milieu. More research is needed to fully understand the direct interaction of prebiotics with immune cells and their signalling events.^35^ Prebiotics like inulin, fructo‐oligosaccharide, β‐glucan, pectin, and xylo‐oligosaccharide (XOS) have gained momentum in various treatment modalities, as seen in Table 2. Their properties and clinical outcome when used as cancer therapeutics have been discussed below.

**TABLE 2:** A summary of the sources and documented health benefits of prebiotics like—inulin, fructooligosaccharide (FOS), β‐glucan, pectin, and xylooligosaccharide (XOS).

Compound	Sources	Benefits	References
Inulin	Chicory root, artichokes, and onions.	They help in promoting the growth of the bacteria present in the gut, improves digestion, reduce inflammation, reduce blood cholesterol level, and increase blood sugar level.	^16^
Fructooligosaccharide (FOS)	Bananas, asparagus, and garlic	Improves gut health, reduces the risk of colon cancer, improves mineral absorption, and enhances immunity.	^31^
Beta‐glucan	Oats, barley, and mushrooms	Reduce cholesterol levels and improve blood sugar control, improve gut health	^12^
Pectin	Apples, pears, and berries	Improve digestion, and reduce inflammation, reduce cholesterol levels and improve blood sugar control.	^26^
Xylooligosaccharide (XOS)	Bamboo shoots, corn cobs, and Sugar cane bagasse	Reduce inflammation, improve mineral absorption, and enhance immune function.	^32^

### 3.1 Inulin

Various forms of fibre have unique physical and chemical characteristics that can all have a varied impact on digestion, controlling hunger, and regulating calorie intake. Particularly, the effects of fermentable fibres on the body, such as inulin‐type fructans (ITFs), are gaining attention.^16^

Several *Arsteracee* sp., including chicory, have roots rich in the carbohydrate inulin, which when hydrolysed by the enzyme inulinase yields a combination of fructose and glucose.^33^ A mixture of oligomers and polymers with 2–60 beta‐2,1‐linked fructose molecules, typically with a 2‐1 linked d‐glucose end, make up this naturally occurring polysaccharide. When inulin is fermented in the colon under anaerobic conditions, it has a prebiotic action that favourably promotes the growth of *Bifidobacteria* sp. in the lower colon.^16^ Moreover, inulin may enhance cardiovascular health, boost the immune system, and improve mineral absorption, among other health advantages. Mice's tumour development, growth, and metastasis were reduced when inulin or oligofructose was added to their diet.

Animal studies have shown that inulin and oligofructose have anti‐carcinogenic and anti‐metastatic properties, which reduce the formation of tumours and enhance the effectiveness of cancer therapies in the treatment of colorectal cancer.^36^ Inulin and oligofructose have the ability to limit the proliferation of cancer cells due to increased levels of *Bifidobacteria* sp. in the colon and their induction of cell wall preparations. The proliferative and apoptotic properties of these two prebiotics are also associated with a reduction in the availability of glucose, a crucial substrate for cancer cells. In addition, a review by Pool‐Zobel on the advantages of ITFs in colorectal cancer listed the following possible mechanisms; lessening exposure to genotoxic agents in the gut or their effects on genotoxicity, growth suppression, gene expression regulation, and/or reducing the ability of colon carcinoma cells to metastasize.^37^, ^38^, ^39^ The anti‐cancer benefits of these non‐digestible carbohydrates are still produced by lowering serum glucose and fatty acid concentrations, which are necessary for the proliferation of cancer cells, according to a few studies on different forms of cancer. Consequences of intestinal flora recovery include advantages of inulin in the prevention of radiation enteritis in gynaecologic cancer patients. The majority of studies in this field only cover probiotics. Patients with pelvic cancer who take probiotics have less diarrhoea brought on by radiation treatment. It appears that the type of probiotics used, and the dosage have an impact on the outcomes.

Moreover, dietary elements may regulate the emergence of hypertension. Certain vegetables include fibre and prebiotics that regulate blood pressure, reducing the onset of hypertension. For instance, inulin is a prebiotic that increases gut health and immune system function especially, by specifically enhancing the growth of *Bifidobacteria* sp. and lowering toxic metabolites. Moreover, the advantages of inulin for metabolic and cardiovascular health are linked to its impact on gut dysbiosis, incretin secretion, dyslipidaemia, and the management of obesity. Short chain fatty acids (SCFA), which are by‐products of inulin fermentation in the gut, have a role in a number of these processes, including the improvement of insulin sensitivity, control of incretin secretion, and reduction of hepatic triacylglycerols.^39^

Intriguingly, SCFA administration, such as propionate, lowers vascular dysfunction and heart hypertrophy in hypertensive mice. Inulin supplementation lowers cardiometabolic risk and boosts antioxidant capacity in diabetic women. Inulin supplementation was reported to lower systolic blood pressure (SBP) and decrease elevations in diastolic blood pressure (DBP) and MAP in breast cancer patients receiving neoadjuvant therapy in several studies that used double‐blind, placebo‐controlled trials.^39^ According to the findings, several trials have demonstrated that dietary fibre supplementation encourages blood pressure reduction, albeit there are some variations related to the timing of supplementation and the change in blood pressure. For instance, consuming fibre for 8 weeks significantly lowers DBP and non‐significantly lowers SBP. In contrast, it was shown that the group supplemented with inulin for 21 days shows a significant reduction in SBP, but not in DBP.^39^ The functions of inulin can be both microbiota dependent and independent, this is because it affects both the host and the gut microbiota. But primarily, inulin functions are dependent on the ability of the microbiota to ferment inulin, which then results into the favourable functions of inulin.

### 3.2 Fructo‐oligosaccharides

Fructo‐oligosaccharides (FOS) are oligosaccharides that naturally occur in a variety of plants, including artichoke, onion, chicory, garlic, asparagus, and banana. They are made up of (2‐1) bonds that connect linear chains of fructose molecules. From 2 to 60 fructose units can be found, and they frequently end with a unit of glucose. The small intestinal glycosidases do not hydrolyse dietary FOS; therefore, it enters the cecum intact. The intestinal bacteria there processes them to produce short‐chain carboxylic acids, l‐lactate, CO_2_, hydrogen, and other metabolites. FOS has a variety of intriguing qualities, such as a low sweetness intensity, as well as being calorie‐free, non‐cariogenic, and classified as soluble dietary fibre.^31^

Moreover, FOS have significant physiological advantages such minimal carcinogenicity, a prebiotic impact, enhanced mineral absorption, and decreased serum levels of phospholipids, triacylglycerols, and cholesterol. Dietary FOS is one of the most widely used commercial prebiotics, and it has long been recognised for its positive effects on consumer health, including immune modulation, betterment of gastrointestinal health and mineral absorption, prevention of colon cancer, and lowered risk of disorders linked to obesity. Dietary FOS are resistant to being hydrolysed by digestive enzymes and maintain their structural integrity until they reach the colon. This study focuses on the prebiotic effects and mechanisms of FOS with a particular emphasis on the possible causal connection between these effects and the positive impacts on health.^31^

A questionable lack of a prevention benefit of dietary fibre has been reported in several reports based on cohort or intervention studies, but new papers, also based on a cohort study, have revealed the opposite finding.^40^ Many studies have found a link between increased dietary fibre intake and a lower risk of colorectal adenomas or colorectal cancer. It is interesting to note that whereas dietary fibres are typically viewed as a whole in research, they actually interact differently with colon microflora individually, which affects their fermentation capabilities and short‐chain fatty acid synthesis. Particularly interesting among these, are fibres that produce substantial amounts of butyrate. In fact, butyrate plays a crucial role in maintaining the colon epithelium's homeostasis. Furthermore, butyrate has been demonstrated to influence the immune system by influencing certain transcription factors. Butyrate‐producing fibres have a preventive effect in animal models of colorectal cancer. Surprisingly, after starch removal, rats do not create significant amounts of butyrate from wheat bran, the fibre utilised in intervention studies, and the establishment of non‐steroidal anti‐inflammatory drug (NSAID) use is one of the ways to tackle colorectal cancer, and it has made progress.^41^, ^42^, ^43^ COX‐2 is a highly inducible gene that is activated by a variety of stimuli, including growth factors and pro‐inflammatory cytokines.

It was found in the study that the combination of FOS and celecoxib (a member of second family) showed reduction in aberrant crypt foci (ACF) which functions as a signature molecule for colon cancer analysis. Another factor taken into account was the quantity of big ACF (4 aberrant crypt per foci), which was referred to as an intermediary biomarker for tumour incidence in rats.^44^, ^45^ The perceived study showed that the administration of celecoxib along with a diet rich in FOS greatly reduced the production of colonic ACF caused by azoxy methane (AOM). This is one of the first account of a COX‐2 selective inhibitor and dietary fibre working together in vivo. The number of ACF, the number of crypts per ACF, and the number of big ACF (4 aberrant crypts per foci) were all reduced by the use of FOS and celecoxib together, indicating that FOS and celecoxib individually have not been able to promote the reduction of ACF, but jointly result in significant decrease in formation of ACF, leading to better control of colon cancer treatment and analysis. The latter parameter's validation as a preliminary biomarker for tumour incidence in the rat model is significant. Celecoxib was chosen as an example of the new class of NSAIDs since it targets COX‐2 specifically and has less negative effects on the gastrointestinal tract than non‐selective NSAIDs.^40^

### 3.3 Beta‐glucan

β‐Glucan are a type of soluble fibres that is found in the cell walls of certain foods such as oats, barley, and mushrooms.^20^, ^46^ They are known to have prebiotic properties, which means that they can selectively stimulate the growth and activity of beneficial bacteria in the gut, which is attributed to their ability to increase the production of SCFAs in the gut. SCFAs can promote the growth of beneficial bacteria and reduce inflammation in the gut, which are important energy sources for colon cells and have been shown to have anti‐inflammatory properties. Additionally, SCFAs can help regulate appetite, lower blood glucose levels, and improve insulin sensitivity.^12^

Prebiotics are non‐digestible fibres that support the growth and activity of probiotics, the beneficial bacteria that live in the gut such as *Bifidobacteria* sp. and *Lactobacilli* sp.^12^ β‐glucan is considered as a prebiotic because it is resistant to digestion in the small intestine and passes through to the colon where it is fermented by gut bacteria. β‐glucan has also been shown to increase the population of beneficial bacteria in the gut, such *Bifidobacteria* sp. and *Lactobacilli* sp., while reducing the population of harmful bacteria.^12^ This can help to improve gut health and reduce the risk of certain diseases, such as inflammatory bowel disease and colorectal cancer. Another health benefit of β‐glucan is lowered cholesterol levels. Studies have shown that β‐glucan can lower cholesterol levels by binding to bile acids in the gut and increasing their excretion, which in turn reduces the amount of cholesterol that is reabsorbed in the body.^47^ β‐glucan is also known to enhance immune function by stimulating the immune system through the activation of immune cells such as macrophages and natural killer cells. This can help defend against infections and diseases. Finally, β‐glucan has been shown to improve blood sugar control by slowing down the absorption of glucose in the gut. This can help regulate blood sugar levels and reduce the risk of diabetes.^48^

Overall, β‐glucan is an important dietary fibre with numerous health benefits. Incorporating beta‐glucan‐rich foods into one's diet, such as oats, barley, and mushrooms, can promote gut health, lower cholesterol levels, enhance immune function, and improve blood sugar control.

### 3.4 Pectin

Pectin is a heteropolysaccharide primarily found in the cell walls of plants. Several trials later, it was concluded that when apple and citrus pectin was combined with the carcinogenic chemical azoxymethane, it resulted in reduced cancer symptoms. It was seen that diets with ample amounts of pectin would support the expression of caspase 1 enzyme and increase the measure of PARPs (Poly (ADP‐ribose) polymerase). Pectin treatments are given if various forms, of which one of the widely known modifications is altered pH. The effect of pectin changes according to the pH of treatment.^23^ Pectin treatments linked with slight base treatment can alter the ester links in the structure of pectin resulting in apoptotic activity. Mostly such active apoptotic pectin is artificially synthesised by increasing the temperature of citrus pectin to about 130°C, which promotes apoptotic response.^26^ A study by Avraham Raz revealed that citrus pectin showed potent anti‐cancer characteristics. Modified citrus pectin (MCP) residues weaken the cell–cell interactions, thereby reducing the number of metastases. MCP also hinders the expression of certain proteins like nm23 which induce metastasis in many cancers. This happens due to greater expression of galactin‐3.^27^

Although pectin is widely used in cancer treatments it also comes with a set of challenges. To overcome the challenges Raj Dutta et al 2013 aimed at developing pectin nanocarriers for targeted drug delivery in cancer therapeutics.^49^ This study focussed on producing encapsulated pectin nanocarriers to target MIA‐PaCa‐2 (pancreas) cancer cell line. They successfully fabricated nanocarriers consisting of superparamagnetic iron oxide nanoparticles and oxaliplatin confined together in a pectin molecule which was linked with Ca^2+^ forming a nanocarriers that could modify pectin magnetically  and targeted drug delivery responses. A superparamagnetic property was well built in MP‐OHP which further had many uses like determination of blocking temperature etc. The physical and chemical properties of pectin were favourable to what they were willing to make. Their aim was to build an oral nanoscale anticancer drug delivery system that would fundamentally target pancreas or the pancreatic cell line responsible for colorectal and pancreatic cancer. Pectin is a versatile polymer and exhibits resistance to protease and amylase, making it easier for MP‐OHP to function. The pH of the molecule was modulated between 5.5 and 7.4 to achieve enhanced permeation and retention in tumours.^24^ Pectin/MCP do not rely on the digestive enzymes to get absorbed into the host's body, they independently get absorbed in the intestines and show anti‐cancer properties.^27^

Another modification in pectin based on enzymatic hydrolysation of citrus pectin was designed which led to the formation of MCP.^27^ MCP is frequently used as a dietary supplement in food products and is regarded as safe by the US FDA. MCP is known for its effective inhibiting properties related to cancer treatments, and therefore it was used in a research study where a prostate cancer cell line was treated.^27^ It was seen that MCP expressed its antineoplastic properties in human prostate carcinoma cells that were tested in various laboratory conditions.^27^ Results showed a significant decrease in the proliferation of tumour cells and a reduction in the invasive behaviour of cancer cells along with induced apoptosis. Studies revealed that a combination therapy approach which included MCP along with ionisation radiation, followed a better cell survival rate.^27^

### 3.5 Xylo‐oligosaccharide

XOS is regarded as a part of functional oligosaccharides that possess prominent prebiotic potential. XOS is believed to have certain beneficial effects on the gut microbiota when its intake is in the form of a dietary supplement.^32^ This can also alter the composition of gut microbes resulting in increased efficacy of cancer therapies. To evaluate the effects of XOS in cancer treatment an experiment was carried out by Aachary in 2015 where contributions of XOS as a prebiotic were examined in colon cancer.^32^ Although XOS is naturally available and can be extracted from a lot of resources like bamboo shoots, it was still prepared from corncob which was pre‐treated with an alkali and then treated with endo xylanase. Results proved that XOS drastically improved the body weight of rats used. The controlled rats were given dimethylhydrazine (DMH) treatment which caused side effects like reduced liver weight that was successfully reversed with the help of XOS supplementation. Due to DMH treatment, there was an adverse effect on the composition of microbes present in the colon. There was a sharp reduction in the population of *Bifidobacteria* sp., and tests revealed an increase in E coli bacteria when compared to normal animals. XOS was supplied orally and infused into the diet to even out this imbalance. Several changes in lipid peroxidation were observed as post‐DMH effects along with decreased activities of enzymes like glutathione‐S‐transferase (GST) and catalase. XOS treatment after DMH administration could reimpose the levels of lipid peroxides in the colon and liver to normal levels.^32^

Recently in 2019, a comparison study was conducted in Korea, which aimed to use XOS as a supplement to symbiotic fermented soymilk to observe its effect on the proliferation of colon cancer. XOS is considered an important prebiotic that functions in enhancing the effects of cancer therapies because of its known for its high nutritional value and the ability to restore gut microbiota. XOS also possesses other strong biological properties. Here, it was complemented with soy milk to check compatibility, side effects of when they are supplied together and benefits of the same. This study first hydrolysed XOS with several enzymes that were isolated from fermented kimchi and then paired it with freshly prepared soymilk. The combination was then sterilised and used on colon cancer cell lines. Prosperous results indicated that there was a rapid increase in the acidification rate and a remarkable reduction in the time of fermentation. Soymilk samples with XOS showed much better viscosity when compared with normal samples. XOS added fibre and prebiotics to the trials which promoted the growth of beneficial bacteria and added to the betterment of protein–polyphenol interactions. The betterment of gut microbiota directly resulted in increased folate synthesis due to XOS supplementation. Apart from this, it was also that XOS expanded the number of viable cell count during storage of the fermented products at 4°C. In cancer cell lines this led to additive anti‐proliferative activities to fight against Caco‐2 and HCT116 cells. It could suppress the signalling pathway in HCT116 cells and did not exert any cytotoxic effects in CCD‐18co cells.^50^ Studies were conducted where XOS was compared with FOS to check the effects of both the oligosaccharides in DMH, fermentation and other treatments. But XOS was clearly a better supplement than FOS and could effectively increase epithelial cell proliferation in precancerous lesions better than FOS. This concludes that XOS has the potential to be used for pre‐cancer lesion treatments as well as it can be supplemented with ongoing cancer therapies as well. The functional activity of XOS is initiated by its fermentation by the gut microbiota in the large intestine of the host. This requires it to pass through the stomach and the small intestine where it gets digested and broken down into various SCFAs that show positive benefits when absorbed.^50^

## 4 PREBIOTICS IN MINIMISING THE DAMAGE DUE TO CHEMOTHERAPY

The human body is home to approximately 39 trillion microorganisms, including various species of bacteria, viruses, and fungi.^51^ The human microbiome plays a significant role in diseases and in health sustenance. It is influenced by various factors including age, lifestyle, genetic makeup, and so on. Any imbalance in the microbiota, also known as dysbiosis, can have major impacts on the human health. It can contribute to life‐threatening diseases, such as cancer and inflammatory bowel disease (IBD), as well as affect major body functions, such as homeostasis and host immunity.^52^

The most common treatment regimen for cancer is chemotherapy which uses cytotoxic drug to kill the rapidly multiplying cancer cells. However, being cytotoxic they have equally disastrous effect on the normal cells as well and can take a toll on the human body in the long run. Though some side effects are mild and tolerable, others can be serious and life‐threatening. Nausea, vomiting, mouth sores, hair loss, skin and nail problems, loss of appetite, fatigue, fever and bowel issues such as constipation and diarrhoea, are some of the prominent side effects of chemotherapy.^53^ Recent studies reveal that chemotherapeutic drugs can also contribute to chemotherapy‐induced gastrointestinal toxicity (CIGT) as they alter the natural balance of the gut microbiota and increase intestinal permeability. Up to 80% of the patients undergoing chemotherapy experience CIGT.^54^ Montassier et al observed a considerable decrease in the diversity of the gut microbiota during a 5‐day high‐dose chemotherapy study. The population of *Firmicutes* sp. and *Actinobacteria* sp. were found to be reduced drastically, whereas those of *Bacteroidetes* sp. and *Proteobacteria* sp. were increased.^55^ This disturbance in the microbial ecosystem of the gut resulting in change of its composition and function is known as gut microbiota dysbiosis. Gut dysbiosis can cause the body to produce more pro‐inflammatory cytokines. These cytokines can change the lining of the gut and cause leaky gut syndrome, which allows bacteria and toxins to pass through the epithelial layer of the gut, into the bloodstream.

Other compounds that have been studied for their prebiotic potential include mannan‐oligosaccharides (MOS), β‐1,4‐mannobiose (MNB), sialyl‐oligosaccharides, xylooligosaccharides (XOS), germinated barley foodstuff (GBF), epilactose, arabinoxylan oligosaccharides (AXOS), and chitooligosaccharides (COS).^56^ Aliyah and As'ad conducted a study to determine the effect of prebiotic supplements on chemotherapy‐induced mucositis (CIM), a common side effect of chemotherapy. This can cause more inflammation and make cancer patients more likely to experience gastrointestinal (GI) problems during treatment.^57^ In addition to physical symptoms, chemotherapy induced gut dysbiosis also causes psychological and cognitive effects in cancer patients and survivors, including depression, anxiety, and post‐traumatic stress disorder.^58^ They observed a considerable improvement in GI symptoms such as nausea, vomiting, constipation, and sore throat.^59^ When prebiotics are fermented by the bacteria in the colon, they produce SCFA. These SCFA, particularly acetic, propionic, and butyric acid, are crucial for maintaining a healthy gut and ensuring proper intestinal function and structure. Butyrate, in particular, provides energy for colon cells and helps to lower the pH in the colon. Lactate also plays a role in promoting gut health by enhancing the immune defence of the gut and increasing the surface area available for absorption.

Maintaining the intestine's barrier function depends heavily on the mucin layer. Mucins build a layer over the epithelial surface, forming a protective mucus blanket that shields the mucosa from bacterial penetration and/or overgrowth. The epithelium is additionally protected from luminal agents like enteric bacterial toxins by mucins, which act as a chemical and physical barrier.^60^ Mucins also produce a wide variety of potential binding sites for bacteria, including pathogenic and commensal bacteria.^61^ As per research, prebiotics interact with their terminal sugars, which bind to the bacteria's adhesion‐promoting receptors, to prevent bacteria from adhering to the intestinal mucin layer.^62^ Additionally, Sinclair et al showed that prebiotics could obstruct cholera toxin's ability to bind to its receptor on the cell, showing that this obstruction is not specific to bacterial cells.^63^ Studies have linked fructooligosaccharides (FOS) and galactooligosaccharides (GOS) to increased mucin production, which reduces bacterial translocation and, in turn, lowers the likelihood of pathogenic infection.^64^, ^65^ Also, by preserving mucosal integrity through mucin alteration, prebiotics has the potential to counteract the damage to the gut caused by chemotherapy. More research is required to learn how prebiotics can affect mucin levels in various intestinal regions in mucositis.

## 5 DISCUSSION AND CONCLUSION

The use of prebiotics as a treatment modality for cancer has been often criticised. Although some translational research has been conducted to assess the efficacy of prebiotics, the long‐term effects on a statistically significant population are yet to be done. As of May 2023, 343 clinical trials involving prebiotics have been successfully completed with only 16 trials being in phase‐4.^66^ However, none of these phase‐4 trials focussed on studying the efficacy of prebiotics as potential cancer therapeutic. This can be attributed to the complex and dynamic nature of gut microbiota interacting with the tumour microenvironment.^67^ An interdisciplinary approach is crucial to understanding the inner workings of these complex surroundings. This has created immense interest in the field of omics and metabolic engineering.^68^

Conditions like chemotherapy‐induced gut dysbiosis can cause several physical, psychological, and cognitive effects in cancer patients and survivors. Prebiotics therapy may offer a promising approach to mitigate these side effects and restore gut health by preventing or reversing dysbiosis, reducing treatment toxicity, and boosting the immune system. More research is needed to fully understand how prebiotics and probiotics work together to improve the immune system, prevent and treat cancer, and reduce treatment symptoms.^4^ Ultimately, improving gut health through prebiotic therapies may provide a novel avenue for managing cancer treatment side effects and improving overall health and quality of life.

## AUTHOR CONTRIBUTIONS

Conceptualization, P.M., V.M.B., S.J., B.N.S. and A.K.; Review of Literature, P.M., V.M.B., S.J., S.S. and S.V.M.; Preparation of Original Draft, P.M., V.M.B., S.J., S.S. and S.V.M.; Reviewing, Editing and Preparation of Final Manuscript, P.M., B.N.S. and A.K.; Administrative and Supervision, B.N.S. and A.K.

## CONFLICT OF INTEREST STATEMENT

The authors declare no conflict of interest.

## ETHICS STATEMENT

No experiments were carried out for this article.

## Data Availability

Data sharing is not applicable to this article as no new data were created or analysed in this study.
